# TGF-β1 Is Present at High Levels in Wound Fluid from Breast Cancer Patients Immediately Post-Surgery, and Is Not Increased by Intraoperative Radiation Therapy (IORT)

**DOI:** 10.1371/journal.pone.0162221

**Published:** 2016-09-02

**Authors:** Sandra D. Scherer, Jochen Bauer, Anja Schmaus, Christian Neumaier, Carsten Herskind, Marlon R. Veldwijk, Frederik Wenz, Jonathan P. Sleeman

**Affiliations:** 1 Centre for Biomedicine and Medical Technology Mannheim, Medical Faculty Mannheim, University of Heidelberg, Mannheim, Germany; 2 Institute of Toxicology and Genetics, Karlsruhe Institute of Technology, Eggenstein-Leopoldshafen, Germany; 3 Department of Radiation Oncology, Universitätsmedizin Mannheim, Medical Faculty Mannheim, Heidelberg University, Mannheim, Germany; Technische Universitat Munchen, GERMANY

## Abstract

In patients with low-risk breast cancer, intraoperative radiotherapy (IORT) during breast-conserving surgery is a novel and convenient treatment option for delivering a single high dose of irradiation directly to the tumour bed. However, edema and fibrosis can develop after surgery and radiotherapy, which can subsequently impair quality of life. TGF- β is a strong inducer of the extracellular matrix component hyaluronan (HA). TGF-β expression and HA metabolism can be modulated by irradiation experimentally, and are involved in edema and fibrosis. We therefore hypothesized that IORT may regulate these factors.Wound fluid (WF) draining from breast lumpectomy sites was collected and levels of TGF-β1 and HA were determined by ELISA. Proliferation and marker expression was analyzed in primary lymphatic endothelial cells (LECs) treated with recombinant TGF-β or WF. Our results show that IORT does not change TGF-β1 or HA levels in wound fluid draining from breast lumpectomy sites, and does not lead to accumulation of sHA oligosaccharides. Nevertheless, concentrations of TGF-β1 were high in WF from patients regardless of IORT, at concentrations well above those associated with fibrosis and the suppression of LEC identity. Consistently, we found that TGF-β in WF is active and inhibits LEC proliferation. Furthermore, all three TGF-β isoforms inhibited LEC proliferation and suppressed LEC marker expression at pathophysiologically relevant concentrations.

Given that TGF-β contributes to edema and plays a role in the regulation of LEC identity, we suggest that inhibition of TGF-β directly after surgery might prevent the development of side effects such as edema and fibrosis.

## Introduction

Radiotherapy is an integral component of cancer treatment, and more than 50% of cancer patients receive radiotherapy during the course of their disease [1]. The dose of irradiation given to the tumour directly correlates with the probability that the tumour will be effectively controlled, yet the dose that can be given is limited by concomitant damage to the surrounding normal tissue. Recent advances in radiation technologies, treatment planning and treatment delivery have resulted in increased sparing of normal tissue outside the clinical target volume. Nevertheless, non-fatal normal tissue responses to irradiation such as edema, fibrosis or chronic pain inside the target volume can significantly impair the patient’s quality of life [2].

To improve the efficacy and reduce the side effects of radiation therapy, intraoperative radiation therapy (IORT) has been developed to deliver radiation directly to the tumour site following surgery [3, 4]. Compared to conventional radiotherapy, a single dose IORT directly delivered to the tumour site yields similar rates of local recurrence and preservation of healthy tissue while at the same time offers the advantages of reduced treatment times [5–8]. Furthermore, IORT can also be used as a boost for patients who receive breast-conserving surgery in combination with external beam radiotherapy (EBRT), allowing any tumour cells remaining after surgery to be subjected to radiotherapy during the surgical procedure, without the necessity of a recovery period [9–11]. However, similar to other radiotherapies, side effects have been reported after IORT, including edema and fibrosis [7, 12–14]. Reducing these side effects offers the promise of further increasing the benefits of IORT.

Fibrosis is typified by the formation of excess fibrous connective tissue, for example during the repair of injured tissue. It is caused by the production of excess extracellular matrix proteins and the accumulation of activated fibroblasts [15]. Transforming growth factor beta (TGF-β) is recognized as being a central regulator of fibrosis [16]. In experimental animals, expression of TGF-β increases in a dose-dependent manner in response to irradiation [17–19]. Consistently, radiation-induced tissue fibrosis is associated with enhanced TGF-β expression in patients [16, 20]. Furthermore, radiation-induced TGF-β expression is functionally involved in the development of radiation-induced fibrosis, as in experimental animals administration of anti-TGF-β antibodies or inhibitors of TGF-β signalling suppressed fibrosis formation subsequent to irradiation [21, 22]. Thus inhibition of TGF-β activity may help to reduce fibrosis after radiation therapy in human patients [20].

Induction of edema in irradiated tissue can compromise the function of the organ concerned, and direct or indirect impairment of the lymphatic vasculature by radiotherapy plays an important role in this pathology [23–25]. Although TGF-β has been reported to promote lymphangiogenesis indirectly in some contexts by stimulating VEGF-C expression in certain epithelial cells [26], evidence has accrued in recent years that the direct effects of TGF-β on lymphatic endothelial cells (LECs) negatively regulate the lymphatic system by suppressing lymphangiogenesis and downregulating expression of genes that determine LEC identity [27, 28]. Furthermore, tissue irradiation results in reduced numbers of lymphatic vessels, lymphatic dysfunction and LEC apoptosis [29, 30]. Notably, blockade of TGF-β activity in an animal model was found to decrease fibrosis and increase lymphatic function after photon irradiation [29]. Together, these data support the hypothesis that radiation-induced TGF-β can contribute to lymphatic dysfunction and thus to the development of edema. Moreover, as TGF-β expression is increased in cells within lymphedematous tissues [31] the development of edema may further exacerbate lymphatic dysfunction.

The glycosaminoglycan hyaluronic acid (HA) is comprised of repeating subunits of N-acetyl glucosamine and glucuronic acid, and is a major component of the extracellular matrix (ECM) [32]. HA is synthesized as a high molecular weight polymer of up to 10^7^ Da (HMW-HA), but during tissue injury and inflammation, enhanced synthesis combined with cleavage of HA, for example through the activities of hyaluronidases and free radicals, can result in HA fragmentation and the accumulation of HA oligosaccharides [33–38]. Ionizing irradiation can also fragment HA directly [39]. Enhanced levels of HA are found in irradiated tissues, consistent with increased expression of hyaluronan synthases in response to irradiation [40, 41]. TGF-β can increase HA synthesis [42, 43] and can also induce expression of the HA synthases HAS1 and HAS2 [44]. Together these observations suggest that irradiation has the potential to stimulate accumulation of small HA (sHA) oligosaccharides (here defined as being of up to 25 disaccharides in length) by increasing both the synthesis and breakdown of HA. We and others have shown that sHA exerts a number of biological effects not observed with HMW-HA, including activation of dendritic cells [45], induction of inflammatory responses [46], angiogenesis [47, 48], lymphangiogenesis [48, 49] and increased expression of matrix metalloproteases and cytokines [50, 51]. Importantly, it has been suggested that HA and TGF-β cooperate during fibrosis [42].

In this study we have addressed the hypothesis that the local edema and fibrosis that can develop after IORT might be caused by increased TGF-β production, increased HA production, and/or accumulation of HA oligosaccharides as a consequence of the irradiation. To this end we examined levels of these factors in wound fluid (WF) draining from the surgical operation sites of breast cancer patients whose primary tumour had been removed, and who had either received or not received IORT. We found that IORT had no significant effect on the levels of these factors in WF. Nevertheless, WF was found to contain substantial levels of TGF-β1 regardless of IORT, at levels well above those associated with fibrosis and impaired lymphatic function. These data therefore suggest that inhibition of TGF-β activity immediately after surgery may be beneficial for breast cancer patients by suppressing the formation of fibrosis and maintaining lymphatic function.

## Materials and Methods

### WF collection and preparation

In this study, 23 patients with low-risk breast cancer were treated with breast-conserving surgery. Of these patients, 11 received intraoperative radiotherapy in addition using a single 20 Gy fraction (50 kV X-ray; Intrabeam, Zeiss, Oberkochen, Germany) as described previously [5, 7]. The age of the patients at treatment without IORT was 66 ±13, whereas the age of patients treated with IORT was 60 ± 12. After surgery, WF was drained from the wound for 24 h as described previously [52]. Thereafter, samples were collected, centrifuged with 800×g for 5 min and the supernatant was filtered through 40 μm filters (BD Falcon, Heidelberg, Germany). After a second centrifugation step (3500×g for 5 min.) the supernatant was subsequently filtered through 5, 0.8 and 0.22 μm filters and aliquots were stored at -80°C.

### Analysis of TGF-β1, total HA and sHA concentrations in WF

TGF-β1 concentrations were quantified using a commercially available ELISA (R&D Systems, Wiesbaden, Germany). Total HA concentrations were analysed using an HA ELISA-like-assay (Echelon, Utah, USA). Concentrations of sHA were determined as recently described [53]. Briefly, WF was centrifuged through Amicon filters with a cut-off of 10 kDa, and sHA concentrations in the ultracentrifugation filtrate were determined using the HA ELISA-like-assay.

### Proliferation assay

Human dermal primary LECs (Promocell, Heidelberg, Germany) were cultivated at 5% O_2_, 5% CO_2_ in EBM-2 MV medium containing growth factors and 5% FCS (Lonza, Basel, Switzerland). LECs were seeded into 96-well plates at a density of 1x10^3^ cells per well and cultured for 24 hours. The cells were then cultivated in the absence of growth factors and FCS for a further 16 hours. For blocking experiments, the cells were pre-treated with 20 μg/ml monoclonal mouse-anti-human TGF-β antibody (R&D Systems, Cat. No. MAB1835) or the corresponding isotype control antibodies (R&D Systems). Cells were incubated for 72 hours with human TGF-β1, -β2, -β3 (Reliatech, Wolfenbüttel, Germany) or with 10% WF from patients. They were then labelled for 16 hours with 1 μCi ^3^H thymidine. To analyze the amount of incorporated radioactivity, the cells were trypsinized for 30 min and harvested onto a glass fibre filter (Wallac, Turku, Finland) using a Harvester 96 cell harvester (Tomtec, Hamden, CT, USA). The filter-immobilised radioactivity was quantified using MicroBeta TriLux Liquid Scintillation together with a luminescence counter (Wallac).

### SDS-PAGE and Western Blot

LECs were treated with human TGF-β1, -β2 and β3 for the indicated time points. Lysates were prepared and subjected to Western blot analysis using 5 μg/ml polyclonal rabbit-anti-human Prox-1 (Reliatech, Cat. No. 102-PA32AG), 1 μg/ml polyclonal goat-anti-human Lyve-1 (R&D Systems, Cat. No. AF2089), 0.2 μg/ml polyclonal goat-anti-human VEGF-R3 (R&D Systems, Cat. No. AF349) and 0.1 μg/ml polyclonal goat-anti-human vimentin (R&D Systems, Cat. No. AF2105) antibodies. For evaluation of protein loading, the blot was probed with 0.01 μg/ml monoclonal mouse-anti-human vinculin-specific antibodies (Sigma Aldrich, Taufkirchen, Germany, Cat. No. V9264). For densitometric evaluation, bands for the Prox-1, Lyve-1, vimentin and VEGFR-3 proteins were normalized to the corresponding loading control using the software ImageJ. Values are presented relative to untreated control samples.

### Ethical standards

Experiments comply with the current laws of Germany. The study was approved by the Medical Ethics Commission II of the Medical Faculty of Mannheim, University Heidelberg and was conducted according to Declaration of Helsinki principles. Specifically, participants were provided with written information about the proposed use of their biological samples, which was also explained to them verbally by the attending physician. Thereafter the participants provided their written consent. This procedure was approved by the ethics committee.

### Statistical analysis

The Kruskal-Wallis test was used to determine whether concentrations of TGF-β1, total HA or sHA were significantly different in WF from patients receiving IORT or not. Statistical significance in proliferation assays was determined using the Student’s t-test.

## Results

**Fig 1:**
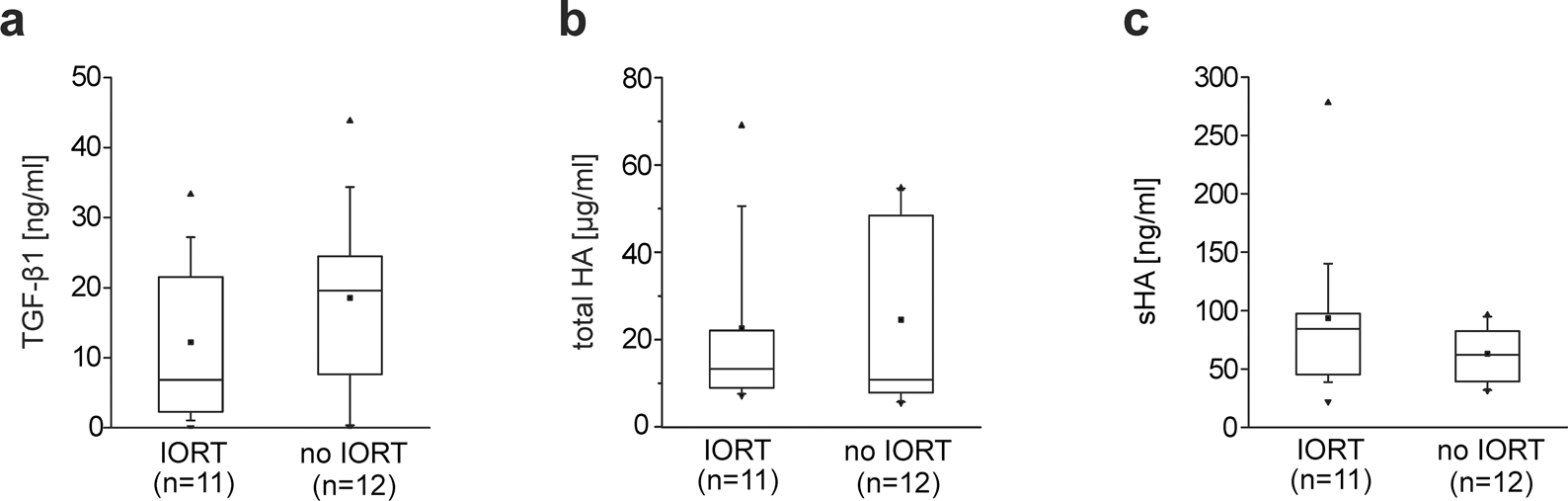
Levels of TGF- β1, total HA and sHA in wound fluid from breast cancer patients with and without IORT. Box-plots comparing the concentrations of TGF-β1 (**a**), total HA (**b**) and sHA (**c**) in interstitial fluid draining from breast lumpectomy sites from patients treated with and without IORT. The upper boundary of the boxes indicates the 75^th^ percentile; the lower boundary indicates the 25^th^ percentile. The line within the boxes indicates the median and the dot within the boxes represents the mean. Whiskers above and below the boxes indicate the 90^th^ and 10^th^ percentile, respectively. The dot lying above the 90^th^ percentile-whisker represents the maximum and the dot below the 10^th^ percentile-whisker represents the minimum. Differences between patients with and without IORT were not statistically significant (Kruskal-Wallis test, TGF-β1 p > 0.18; total HA p > 0.49; sHA p > 0.06).

To determine if IORT can lead to the production and accumulation of TGF-β1, HA or sHA, WF draining breast lumpectomy sites from breast cancer patients treated or not treated with IORT during surgery was collected. Levels of TGF-β1, HA and sHA were measured in the WF. IORT had no significant effect on the concentration of any of these factors (Fig 1). The volume of WF draining from the surgical site was comparable in patients without IORT (39.9 ml ± 39.9) or treated with IORT (34.8 ml ± 34.7). As the volume of WF draining from the surgical site differed widely from patient to patient, we determined whether the concentrations of TGF-β1, HA or sHA in WF correlated with the volume of WF. No correlation between the concentration of TGF-β1 and the volume of WF collected (correlation coefficient 0.00227) was observed. Similar results were obtained for HA and sHA levels (correlation coefficients 0.00361or 0.0064, respectively). These results rule out the possibility that differences in the volume of WF account for variance in the concentrations of TGF-β1, HA or sHA in WF.

**Fig 2:**
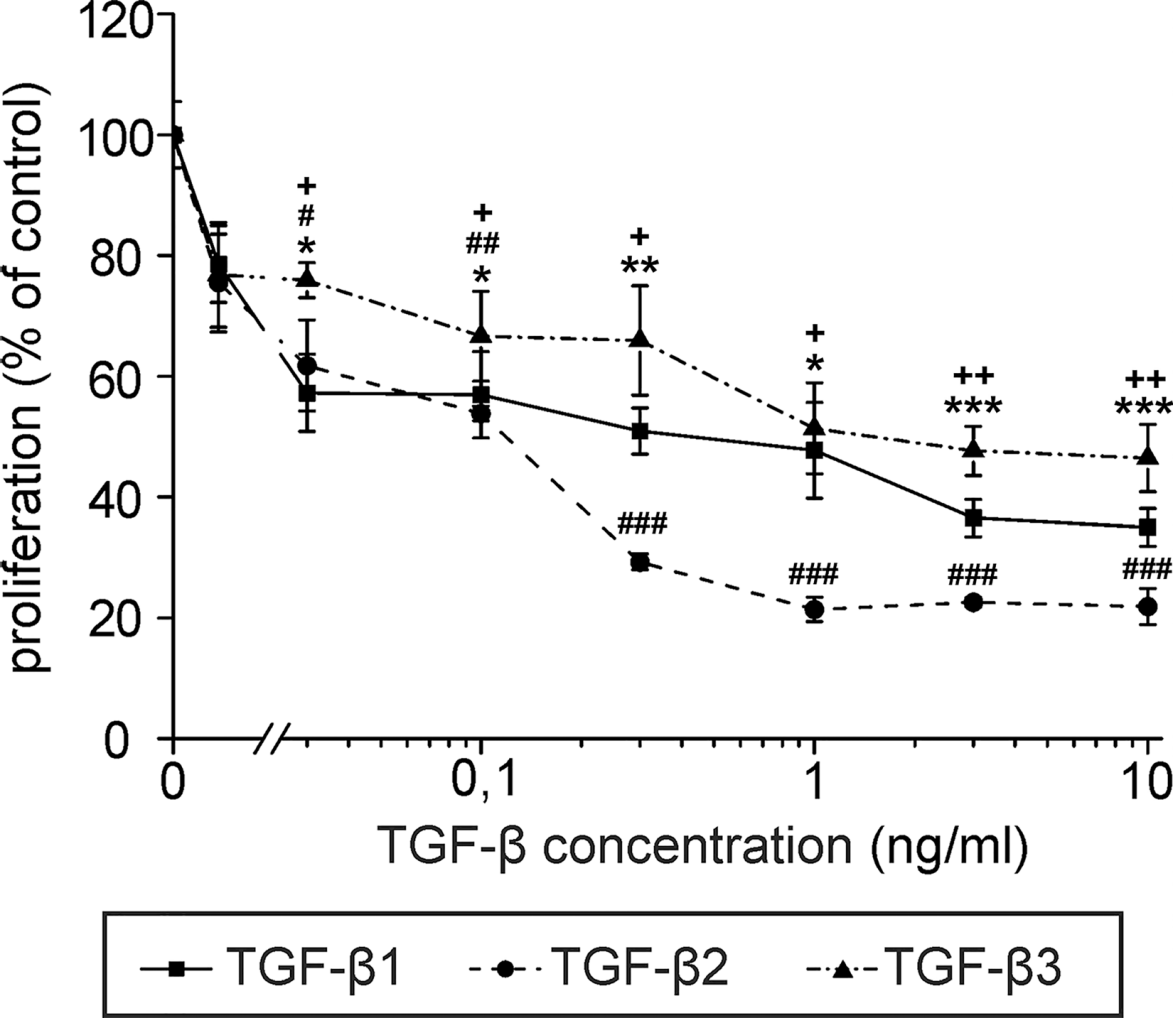
TGF-β1, -β2 and -β3 inhibit the proliferation of LECs. Primary human LECs were incubated with different concentrations of recombinant TGF-β1, -β2 or -β3 or were left untreated and served as control. Proliferation was assessed using ^3^H thymidine incorporation assays. Data represent the mean of triplicate samples, error bars indicate +/- standard error. Student’s t-test: TGF-β1 relative to untreated control: * p < 0.05, ** p < 0.005, *** p < 0.001; TGF-β2 relative to untreated control: # p < 0.05, ## p < 0.005, ### p < 0.001; TGF-β3 relative to untreated control: + p < 0.05, ++ p < 0.005, +++ p < 0.001. The experiment shown is representative of three independent replicates.

Strikingly, high levels of TGF-β were found in the WF of both groups of patients, with concentrations up to more than 40 ng/ml, and mean values between 10–20 ng/ml (Fig 1A). As TGF-β has been reported to suppress lymphangiogenesis and lymphatic identity [27, 28], we determined whether the concentrations of TGF-β in WF influence LEC proliferation and expression of lymphatic markers. To this end, the proliferation of primary LECs treated with different concentrations of recombinant TGF-β1, -β2 and -β3 was measured. All three TGF-β isoforms significantly inhibited LEC proliferation at concentrations of TGF- β1 found in WF (Fig 2).

**Fig 3:**
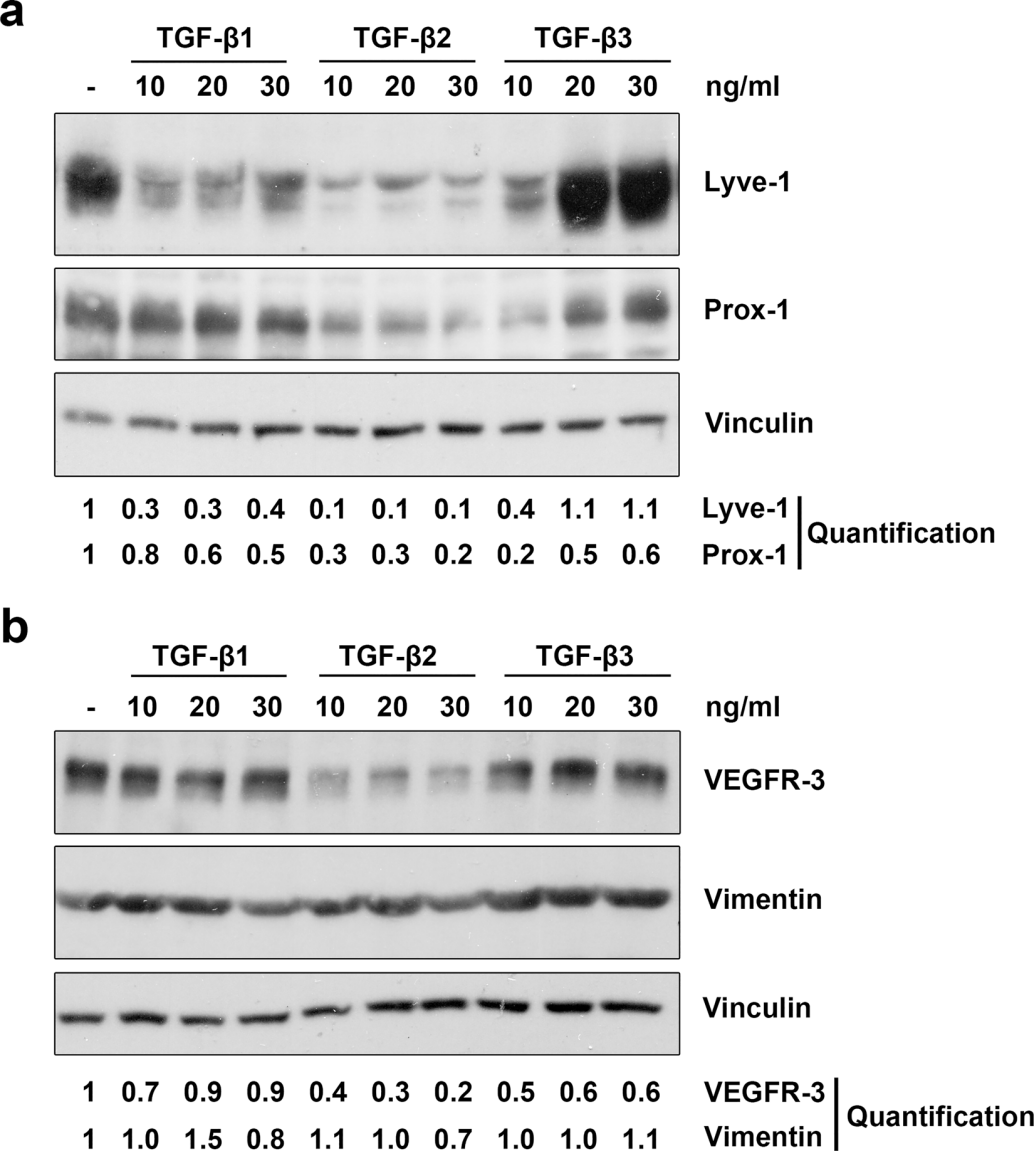
TGF-β1, -β2 and -β3 reduce lymphatic marker expression in LECs. Primary human LECs were treated with 10, 20 or 30 ng/ml TGF-β1, -β2 and -β3 for 72 hours (**a**) or 100 hours (**b**). Untreated cells served as a control. Lysates were prepared and analysed by Western blot using antibodies specific for Lyve-1, Prox-1, VEGFR-3 or vimentin. Vinculin served as loading control. The experiment was performed twice with equivalent results. For densitometry evaluation, protein bands were analysed using the software ImageJ. Bands for the Prox-1, Lyve-1, vimentin and VEGFR-3 proteins were normalized to the corresponding loading control and are displayed as the expression level relative to the untreated control samples.

Furthermore, we treated primary LECs with different concentrations of recombinant TGF-β1, -β2 and -β3 and analysed the expression of different lymphatic markers. Concentration-dependent and isoform-specific effects were observed (Fig 3). TGF-β1 reduced expression of Lyve-1, while TGF-β2 potently suppressed expression of Lyve-1, Prox-1 and VEGFR-3. Interestingly, TGF-β3 reduced the expression of Lyve-1 and Prox1 at 10 ng/ml, but higher concentrations of between 20–30 ng/ml induced expression. None of the TGF-β isoforms influenced the expression of vimentin, suggesting that the cells do not acquire a mesenchymal phenotype as a consequence of TGF-β treatment.

**Fig 4:**
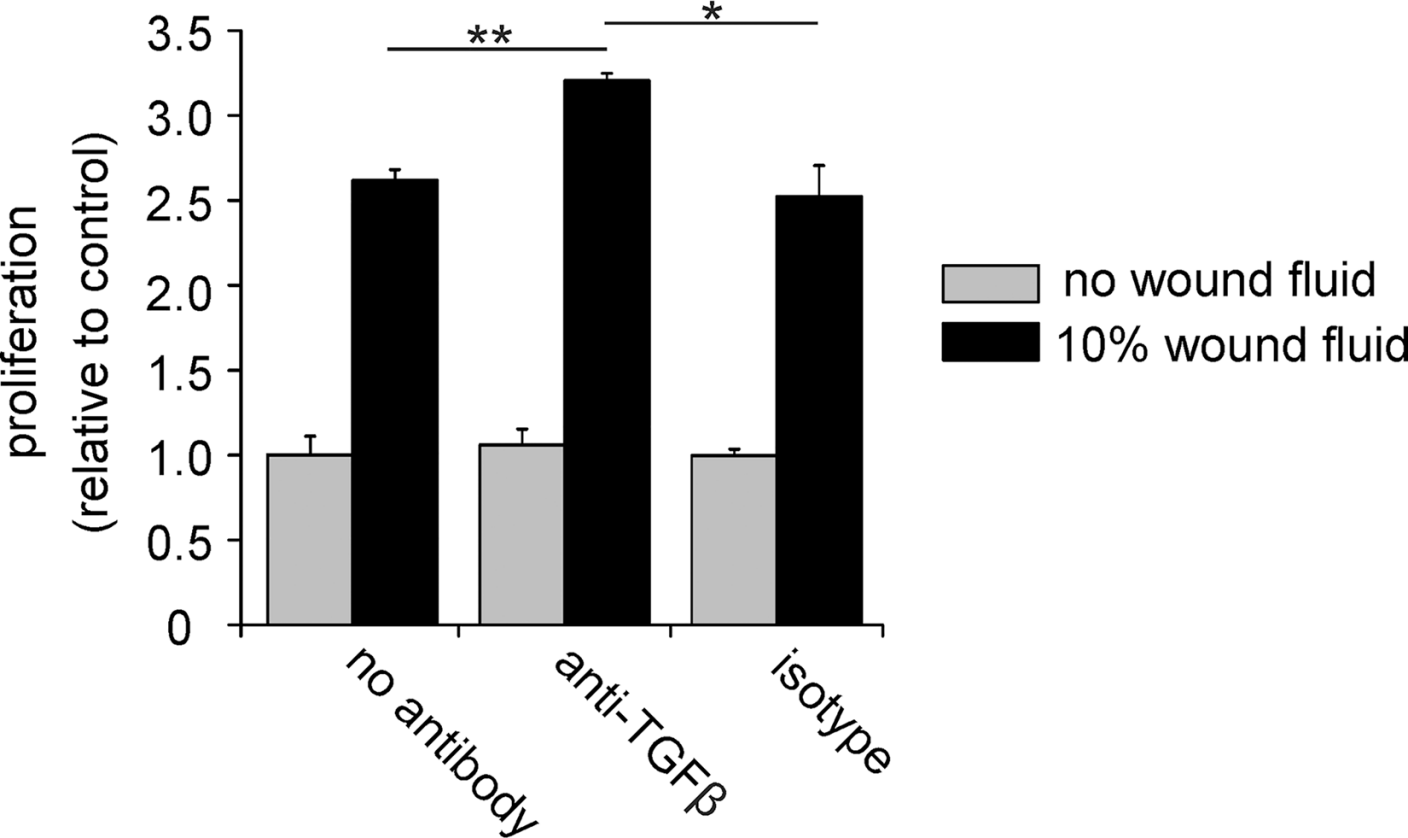
TGF-β in wound fluid is active and inhibits LEC proliferation. Primary human LECs were incubated with or without 10% wound fluid from a patient and their proliferation measured using ³H thymidine incorporation. The mean and SE of triplicate samples is shown. Pretreatment with neutralizing TGF-β antibodies increases wound fluid induced LEC proliferation. Student’s t-test: * p < 0.05, ** p < 0.005.

To check if TGF-β present in WF is biologically active, we treated primary LECs with WF and measured their proliferation in the presence or absence of neutralizing anti-TGF-β antibodies. Neutralization of TGF-β activity in the WF significantly increased the proliferation rate of the cells in response to WF (Fig 4), indicating that TGF-β is active and suppresses LEC proliferation in the context of WF.

Together these data show that TGF-β1, HA and sHA are present in WF draining from breast cancer surgical sites but that their levels are not influenced by IORT. Our data also reveal that all TGF-β isoforms suppress LEC proliferation and modulate expression of markers of lymphatic identity at concentrations present in WF.

## Discussion

Here we report that IORT during breast cancer surgery does not affect levels of TGF-β1, HA and sHA in fluid draining from the operative site. Nevertheless, high levels of TGF-β1 are present in WF, regardless of IORT. These levels inhibit proliferation of LECs and suppress lymphatic identity by down-regulating key LEC markers. We conclude that inhibition of TGF-β activity following surgical removal of breast cancer may be beneficial for patients by suppressing edema caused by TGF-β-mediated lymphatic dysfunction, and by reducing fibrosis and scarring.

IORT was established with the aim of delivering a higher effective radiation dose to tumour sites. Clinical studies have reported that IORT has a similar efficacy and tolerance compared to conventional EBRT [14] and has a high acceptance by patients [54]. Our finding that IORT does not alter levels of TGF-β1, HA or sHA provides further evidence for the efficacy of IORT, as no additional side effects due to enhanced production of these highly biologically active molecules are likely to be induced.

The concentration of both total HA and sHA we found in the WF is similar to the levels we have previously reported in interstitial fluid from normal tissues [53]. Thus although irradiation can increase HA synthesis [40] and directly fragment HA, IORT appears to have no effect on HA synthesis and breakdown, at least during the first 24 hours following surgery. Importantly, these data suggest that direct fragmentation of HA by therapeutic irradiation does not lead to accumulation of biologically active sHA oligosaccharides in the milieu of the irradiated tissue, and therefore potential tumour-promoting effects of sHA accumulating as a consequence of irradiation that could foster recurrence can be discounted.

The data in Fig 4 suggest that as yet undefined factors in WF can stimulate lymphatic endothelial cell proliferation. Consistently, high concentrations of growth factors and cytokines were found in fluid draining subcutaneous wounds [55] and in WF from donor sites for split-thickness skin grafts [56].

To the best of our knowledge, this is the first study to report TGF-β1 levels in fluid draining from wounds following surgical removal of tumours. Notably the concentrations of TGF-β1 we found are considerably higher than those previously reported in fluid draining subcutaneous wounds [55], from donor sites for split-thickness skin grafts [56] and from burn blister fluid [57]. Higher levels of TGF-β1 were observed in the WF draining tumour excision sites compared to other types of wounds. Consistently, enhanced levels of TGF-β are detected in the blood of breast cancer patients compared to healthy controls, and these levels diminish rapidly following surgery to normal levels [58–60]. The increased levels of TGF-β found in fluid from subcutaneous wounds also decay after 24–48 hours [55]. Although we only investigated the concentrations of TGF-β1 in WF 24 hours after surgery, these observations suggest that high levels of TGF-β1 in the post-excision wound are likely to be transient.

The functions of TGF-β isoforms often overlap, for example in the induction of fibrosis [61]. However, there are also marked differences in their roles, as exemplified by the phenotype of the individual knockout mice [62, 63]. With regard to the regulation of lymphangiogenesis, TGF-β1 has previously been identified as an anti-lymphangiogenic growth factor [27, 28]. Our data extend these observations by demonstrating that not only TGF-β1 but also TGF-β2 and -β3 can inhibit the proliferation of primary LECs with a similar potency. Nevertheless, at concentrations of TGF-β1 present in WF, the three isoforms have distinct effects on lymphatic marker expression, with TGF-β2 exhibiting the most pronounced suppression of lymphatic identity, and TGF-β3 showing a concentration-dependent biphasic inhibition and increase of marker expression. This suggests that the function of lymphatic vessels and lymphatic clearance *in vivo* might depend on the relative presence of the individual TGF-β isoforms. Although we analysed here only the concentrations of TGF-β1 in the fluid draining lumpectomy sites, TGF-β1 is likely to be a predominant isoform because WF from donor sites for split-thickness skin grafts contains around 15-fold higher levels of TGF-β1 compared to TGF-β2 [56]. Similarly, more than 50-fold higher levels of TGF-β1 compared to TGF-β2 have been reported in burn blister fluid [57].

The high TGF-β1 levels we found in WF are sufficient to induce fibrosis and impair lymphatic function [27, 28], suggesting that TGF-β1 in the milieu of the operative site may significantly contribute to the formation of edema and fibrosis observed after breast lumpectomy. Inhibition of TGF-β immediately after surgery, for example locally, may therefore be beneficial for breast cancer patients by suppressing scarring and improving lymphatic drainage. While long-term anti-TGF-β may lead to unacceptable side effects [64], short-term TGF-β inhibition immediately following eye surgery has been shown to have clinical utility in suppressing scarring [65]. Our results suggest that similar approaches may prove beneficial following breast cancer surgery, particularly because high TGF-β levels are likely to be transient (see above).

Pre-clinical studies in animal-models support the notion that interfering with the TGF-β pathway holds promise for the treatment of edema and fibrosis. Sequestering TGF-β with soluble TGF-β receptor molecules prevents abnormalization of lymphatic vessels and improves lymphatic drainage in a murine ovarian carcinoma model [66]. In a mouse lymphedema model, blocking TGF-β improved lymphatic function, and reduced edema and fibrosis formation [31]. In bleomycin-induced mouse models of skin fibrosis, interfering with the TGF-β pathway using peptide 144, which blocks the interaction between TGF-β1 and TGF-β1 type III receptor, reduced fibrosis formation [67]. In addition, interfering with the TGF-β pathway can diminish the tissue damage that occurs after irradiation, for example by improving lymphatic function, and by reducing tissue fibrosis, morphological changes and inflammatory responses [21, 22, 29]. Consistent with the observations, we show here that inhibition of TGF-β augments WF-induced LEC proliferation.

A number of agents that interfere with TGF-β signalling are being clinically evaluated in the context of fibrosis and tumour treatment [68, 69], including neutralizing antibodies that abolish ligand receptor interactions, recombinant Fc-fusion proteins containing receptor ectodomains to sequester TGF-β, small molecule inhibitors of the TGF-β receptor kinases, and antisense oligonucleotides that reduce TGF-β expression. Fresolimumab (GC1008), a pan-TGF-β neutralizing antibody has been used in clinical trials to treat patients with a variety of malignancies and with different forms of fibrosis [70]. Blocking TGF- β signalling has been suggested to reduce radiation side effects and will be tested in a clinical trial using Fresolimumab in combination with stereotactic ablative radiotherapy in NSCLC (clinicaltrials.gov NCT02581787). In systemic sclerosis patients, treatment with Fresolimumab was able to reduce skin fibrosis [71]. Trabedersen (AP12009), antisense oligonucleotides that reduce expression of TGF-β2, have been tested in a phase IIb study in glioma patients, and showed promising results concerning efficiency and safety [72]. Furthermore, Galunisertib (LY2157299), a small molecule inhibitor selective for the kinase domain of TGFβ receptor 1 that specifically downregulates the phosphorylation of SMAD2, showed anti-tumour effects in a first clinical trial [73] and is currently being tested in phase 2 in glioblastoma patients and in patients with myelodysplastic syndromes [69].

In conclusion, this study shows that high levels of active TGF-β1 are present in the milieu of post-operative breast lumpectomy sites. Together the results suggest that inhibition of TGF-β activity might not only be considered for anticancer therapy, but could also be useful for the prevention and treatment of the side effects of therapy, such as edema and fibrosis.
